# Abnormal Striatal-Cortical Networks Contribute to the Attention/Executive Function Deficits in Idiopathic REM Sleep Behavior Disorder: A Resting State Functional MRI Study

**DOI:** 10.3389/fnagi.2021.690854

**Published:** 2021-07-01

**Authors:** Hong-Ju Zhang, Sheng-Hui Wang, Ying-Ying Bai, Jie-Wen Zhang, Shuai Chen

**Affiliations:** Department of Neurology People’s Hospital of Zhengzhou University, Zhengzhou, China

**Keywords:** REM sleep behavior disorder, striatum, functional MRI, cognition, functional connectivity

## Abstract

**Introduction:**

The structural and functional damages of the striatum were evident in idiopathic REM sleep behavior disorder (iRBD). With the research on iRBD deepens, cognitive impairment in iRBD is getting increasing attention. However, the mechanism of cognitive impairment in iRBD was poorly understood.

**Methods:**

Neuropsychological assessment was carried out in 21 polysomnographies (PSGs) confirmed iRBD patients and 22 normal controls. Both regional homogeneity (ReHo) and seed-based functional connectivity (FC) rs-fMRI analyses were applied to explore the FC abnormalities and its association with cognition in iRBD patients. Positive ReHo clusters were set as seeds for further FC analysis.

**Results:**

Idiopathic REM sleep behavior disorder patients presented cognitive deficits in attention/working memory, executive function, immediate memory, and visuo-spatial ability. ReHo analysis revealed abnormal spontaneous brain activities in the striatum (right caudate, left pallidum and bilateral putamen) in iRBD. FC analysis showed decreased striatum-related FCs in the frontal, temporal, occipital lobes, thalamus, anterior cingulate gyrus, as well as decreased intrinsic FCs between bilateral putamen and between caudate and pallidum. Deficits in attention/working memory, executive function, and immediate memory were associated with abnormal striatal-cortical FCs including frontal, temporal, and anterior cingulate cortices.

**Conclusion:**

Functional changes of striatum and cognitive impairment in iRBD were reconfirmed in the present study. Abnormal striatal-cortical networks, especially the striatal-frontal network, contribute to the working memory/executive function deficits in iRBDs. These findings supported the role of striatum not only in motor but also in cognition impairment in iRBD.

## Introduction

REM sleep behavior disorder (RBD) is characterized by increased muscle tone in REM sleep, accompanied by dreams and abnormal limb movements. In recent years, more and more attention has been paid to RBD because of its strong relationship with a variety of neurodegenerative diseases. RBD can present as a non-motor symptom in α-synucleinopathies including Parkinson’s disease (PD), dementia with Lewy bodies (DLB), and multiple system atrophy (MSA). In addition, idiopathic RBD (iRBD) is also the most valuable predictor for future development of α-synucleinopathies (Hu, 2020). According to the study by the international RBD study group, RBD is converted into neurodegenerative diseases with an annual probability of 6.3% (Postuma et al., 2019). A meta-analysis of longitudinal studies on iRBD found the risk of developing neurodegenerative diseases was 33.5% after 5 years follow-up, 82.4% at 10 years, and 96.6% at 14.9 years. Among these neurodegenerative diseases, 43% were PD and 25% were DLB (Galbiati et al., 2019).

Besides abnormal sleep behavior, iRBD can also present some similar clinical features seen in PD, such as cognitive impairment, olfactory impairment, constipation, depression, decreased striatum dopamine transporter on SPECT, and hyperechogenicity in substantia nigra. With the research on iRBD deepens, cognitive impairment in iRBD is getting increasing attention. Nearly 33–50% of iRBD patients had mild cognitive impairment (MCI) (Gagnon et al., 2009; Zhang et al., 2016). The risk of developing future dementia also increased in iRBD with MCI (Postuma et al., 2019). Several neuropsychological studies revealed cognitive decline in attention, executive function, memory and visuo-spatial domains in iRBD patients (Massicotte-Marquez et al., 2008; Marques et al., 2010; Gagnon et al., 2012; Plomhause et al., 2014; Assogna et al., 2021). Although the affected domains in each study were not completely consistent, impairment of attention, executive function, and visual-spatial abilities seemed to be prominent (Ferini-Strambi et al., 2019). Besides, among all cognitive domains, attention and executive dysfunction were found correlated with phenoconversion of iRBD (e.g., iRBD to PD or DLB) in longitudinal studies (Youn et al., 2016).

Unlike PD, the mechanisms of cognitive impairment in iRBD were not well studied. In PD, the underlying mechanisms of cognitive impairment in each domain may be different. From the perspective of neural networks, attention/executive dysfunction in PD may be closely related to the striatum-frontal dopaminergic network, while memory and visuo-spatial dysfunction may be related to the posterior cortex with the pathological basis of Lewy body and/or Aβ deposition (Bailey and Goldman, 2017). Various neuroimaging methods have found that, similar to PD, iRBD patients can have similar structural and functional abnormalities in the regions of brain stem, striatum, and cortex (Ferini-Strambi et al., 2019). As prodromal PD, it seems reasonable that cognitive impairment in iRBD may be similar to PD.

Resting state functional magnetic resonance imaging (rs-fMRI) is a non-invasive method using blood oxygenation level-dependent fMRI (BOLD-fMRI) to investigate spontaneous brain activity. The analyses of regional homogeneity (ReHo) and functional connectivity (FC) are useful methods in identifying brain network activity changes in neurodegenerative diseases (Pievani et al., 2011). One study detected abnormal spontaneous brain activity of the bilateral putamen in iRBD patients (Li et al., 2020). By setting seed in substantia nigra (SN), iRBD showed abnormal FCs in nigrostriatal and nigrocortical networks (Ellmore et al., 2013). Using a pre-established template of basal ganglia network, another study discovered altered basal ganglia connectivity in iRBD, similar to that in PD (Rolinski et al., 2016). However, research of cognitive impairment in iRBD utilizing resting state fMRI was rare.

To explore the underlying mechanism of cognitive impairment in iRBD, the current study used both ReHo and seed-based FC approaches to explore the FC abnormalities and its association with cognitive function in iRBD patients.

## Subjects and Methods

### Participants

Twenty-one patients with iRBD were recruited as the iRBD group at Department of Neurology, People’s Hospital of Zhengzhou University from December 2015 to 2018. The diagnosis of all iRBD patients were confirmed by polysomnography according to the third edition of the International Classification of Sleep Disorders (ICSD-3). The exclusion criteria were secondary RBD, such as intracranial structural abnormalities, other sleep disorders, PD-associated RBD, and drug-induced RBD; history of neuropsychiatric diseases, brain trauma, and cranial surgery; history of long-term use of psychiatric drugs and substance abuse. Twenty-two age and gender-matched healthy controls (HCs) were recruited as the HC group. Written informed consent for clinical and neuroimaging data collections were obtained from all subjects. The study was approved by the Medical Ethics Committee of People’s Hospital of Zhengzhou University (No:201705).

### Neuropsychological Assessment

We examined the cognitive performance of 21 patients with iRBD and 22 normal controls. On neuropsychological testing, global cognition was assessed with Mini-mental State Examination (MMSE) (Katzman et al., 1988). Attention/working memory domain was assessed by digit ordering test (DOT) and trail making test A (TMT-A) (Hoppe et al., 2000; Zhao et al., 2013), executive function by trail making test B (TMT-B) and symbol digital modalities test (SDMT), visuo-spatial ability by rey-osterrieth complex figure test (ROCFT-copy) (Shin et al., 2006), and memory domain by auditory verbal learning test-H (AVLT-H) (Guo et al., 2009). AVLT-H measured the recall of 12 words after three learning trials. N1,2,3 was for immediate recall during the learning phase (which is closely related to attention/working memory); N4 was for short delayed recall at 5 min; N5 was for delayed recall at 30 min; N6 was for category-cued recall. For the MMSE, TMT, DOT, ROCFT-copy, and AVLT-H tests, higher raw scores correspond to better cognitive performance, whereas for the TMT tests, higher scores correspond to a worse cognitive function.

### MRI Data Acquisition and Pre-processing

Magnetic resonance imaging data were acquired by a 3.0 T GE Discovery MR 750 scanner (General Electric, Fairfield, CT, United States) with an eight-channel head-neck coil at People’s Hospital of Zhengzhou University. Head motions were controlled and foam pads were used to diminish scanner noise. All subjects wearing dark eye masks and ear plugs were instructed to relax, keep their eyes closed, and think of nothing or fall asleep during the acquisition. In order to exclude structural brain abnormalities, a high-resolution anatomical scan was acquired using a 3D fast spoiled gradient recalled (FSPGR) sequence with the following parameters: TR = 8.2 ms, TE = 3.22 ms, matrix size = 256 × 256, slices = 156, slice thickness = 1.0 mm, FOV = 240 mm, and FA = 12°. Resting-state functional MR data were acquired using a standard GRE-EPI sequence with the following parameters: TR (repetition time) = 2,000 ms, TE (echo time) = 30 ms, TR (reverse time) = 450 ms, FOV (field of view) = 240 mm, matrix size = 64 × 64, slices = 39, slice thickness = 4 mm, and FA (flip angle) = 90°.

Data pre-processing were conducted using DPARSF^1^, based on Statistical Parametric Mapping software SPM8^2^ and REST^3^ in MatLab platform (Version 2013a, MathWorks Inc., Natick, MA, United States). The first ten scans of rs-fMRI images were discarded for magnetization equilibrium. The remaining consecutive images underwent slice-timing correction for reducing the errors of acquisition, and head-motion correction for eliminating the impact of head movement on datasets. None of the subjects had head motions exceeded 3 mm or 3.0 for translation or rotational parameters. Following, the images were spatially normalized to the Montreal Neurological Institute 152 template in order to reducing individual differences, and then, resampled to 3 mm cubic voxels. The images were linearly detrended and temporally bandpass-filtered (0.01–0.08 Hz) to eliminate low-frequency drift and high-frequency physiological noise. Finally, global mean signal, white matter signal, cerebrospinal fluid signal, and six motion parameters were regressed out. For FC analysis, datasets were spatial smoothed using an isotropic 4 mm full-width at half-maximum (FWHM) Gaussian spatial kernel in order to remove image noise.

### Regional Homogeneity Analysis

Individual ReHo maps were generated by calculating the Kendall coefficient of concordance (KCC) between the time series of a given voxel and its nearest neighbors in a voxel-wise way using DPARSF. To minimize individual variation in KCC value, normalization of the ReHo maps was done by dividing the KCC for each voxel by the average KCC for the whole brain. Then, ReHo maps were spatially smoothed. Voxel-by-voxel ReHo comparisons were applied between iRBD and HC using two-sample *t*-test in SPM8 with age, gender, education years, and head motion parameters (mean FD_Jenkinson) adjusted. Threshold value was set at *P* < 0.05 using Gaussian random field (GRF) correction for statistical analysis.

### Functional Connectivity Analysis

After obtaining the abnormal ReHo regions, that were the right caudate, left pallidum and bilateral putamen, we used these ReHo regions as seeds for further FC analysis. The mean time course from each seed was extracted by averaging the time series of all voxels within the seed. Then, Pearson’s correlation was calculated between the averaged time series for each seed and the rest of the brain, and obtaining a *r*-value function connectivity (r-FC) map. Finally, in order to acquire an approximately normal distribution for r-FC, Fisher’s z transformation was applied to convert r-FC map into z-FC maps for each individual. z-FC comparisons between iRBD and HC were conducted by two-sample *t*-test adjusting for age, gender, education, and head motion parameters. A Threshold-Free Cluster Enhancement (TFCE) corrected threshold of *P* < 0.05 was set as statistically significant with cluster size >10 voxels.

### Statistical Analysis

Categorical variables were analyzed with Chi-square test. For continuous variables, the Kolmogorov–Smirnov test was used to test for normality. Student’s *t*-test was used for continuous variables with normal distribution. Mann–Whitney *U*-test was used for variables not normally distributed. Pearson partial correlations were used to analyze the correlations between abnormal FCs and cognitive performance scores after adjusting for age, sex, and education. This study mainly investigated whether the basal ganglia-cortical network correlated with cognitive impairment, especially the working memory/executive function. Although multiple comparisons were done between FC abnormalities and cognitive performance, yielding some statistically significant results, several comparisons were beforehand known to have no theoretical basis. Besides, the sample size was small, and cognitive subtests were numerous. For these reasons, we did not perform corrections for multiple comparisons to avoid type II errors. The significant threshold value set at *P* < 0.05 for all statistical analysis. All the statistical analyses were done by SPSS19.0 (SPSS Inc., Chicago, IL, United States).

## Results

### Demographic and Clinical Characteristics

Twenty-one patients with iRBD were recruited with the mean onset age of 60.6 years and mean duration of 3.6 years. None of the iRBD patients took drugs that might affect RBD, such as SSRIs, SNRIs, and benzodiazepines, in the past 6 months before enrollment. No significant differences were observed for age, gender, and education years between the iRBD group and HC group. Compared to HC, iRBD had significant lower scores in ROCFT_copy, AVLT-N1, AVLT-N2, AVLT-N3, AVLT-N1-3 (immediate memory), SDMT, and DOT and greater scores in TMT-B (*P* < 0.05; Table 1). There were no significant differences in MMSE scores, AVLT-N4 (short delayed recall), AVLT-N5 (long delayed recall), AVLT-N6 (cued recall), AVLT-N1-6 (sum of six trials), and TMT-A between the two groups (*P* > 0.05; Table 1). Thus, ROCFT_copy, AVLT-N1-3, TMT-B, DOT, and SDMT were chosen for the subsequent association studies.

**TABLE 1:** Demographic and clinical characteristics between iRBD and control groups.

Characteristics	iRBD (*n* = 21)	CON (*n* = 22)	*P* value
Age *M* (P25, P75), years	64.00 (57.50, 67.00)	60.00 (57.25, 65.25)	0.463^c^
Gender M/F	14/7	9/13	0.091^b^
Education mean ± SD, years	8.81 ± 3.40	8.86 ± 3.14	0.957^a^
MMSE mean ± SD, score	27.33 ± 1.39	27.32 ± 1.21	0.970^a^
ROCFT _copy *M* (P25, P75), score	32.00 (30.50, 34.00)	34.50 (32.00, 36.00)	**0.016**^c^
AVLT-N1 mean ± SD, score	3.86 ± 1.82	5.00 ± 1.23	**0.020**^a^
AVLT-N2 mean ± SD, score	5.29 ± 1.88	6.64 ± 1.14	**0.006**^a^
AVLT-N3 *M* (P25, P75), score	6.00 (5.00, 8.00)	7.50 (6.00, 9.00)	**0.017**^c^
AVLT-N4 *M* (P25, P75), score	6.00 (3.50, 8.00)	6.00 (6.00, 8.00)	0.139^c^
AVLT-N5 *M* (P25, P75), score	7.00 (3.00, 7.00)	6.00 (5.00, 6.00)	0.824^c^
AVLT-N6 M (P25, P75), score	6.00 (4.50, 8.00)	6.00 (5.00, 7.00)	0.824^c^
AVLT-N1-3 mean ± SD, score	15.57 ± 5.33	19.32 ± 3.55	**0.009**^a^
AVLT-N1-6 mean ± SD, score	32.29 ± 12.21	38.09 ± 6.91	0.061^a^
SDMT *M* (P25, P75), score	22.00 (20.00, 31.00)	32.50 (28.75, 36.00)	**0.000**^c^
TMT-A test *M* (P25, P75), seconds	86.00 (58.00, 110.00)	63.00 (49.50, 85.00)	0.055^c^
TMT-B test *M* (P25, P75), seconds	192.00 (144.50, 210.00)	116.00 (99.75, 197.25)	**0.024**^c^
DOT *M* (P25, P75), score	5.00 (4.00, 5.00)	5.50 (5.00, 6.00)	**0.029**^c^
MDS-UPDRS-III *M* (P25, P75), score	0.00 (0.00, 2.00)	0.00 (0.00, 1.00)	0.000^c^

*iRBD, idiopathic rapid eye movement sleep behavior disorder; HC, healthy controls. ROCFT, rey-osterrieth complex figure test; AVLT, auditory verbal learning test; SDMT, symbol digit modalities test; TMT, trail making test; DOT, digit ordering test; MDS-UPDRSIII, movement disorder society unified Parkinson’s Disease rating scale motor section. Data are presented as mean ± standard deviation, or median (interquartile range) as appropriate. ^a^Student’s test for two groups. ^b^Chi square test for two groups. ^c^Mann–Whitney *U*-test for two groups. For the comparisons of cognitive tests, P < 0.05 are shown in bold.*

### Different ReHo Regions Between iRBD and HC

**FIGURE 1:**
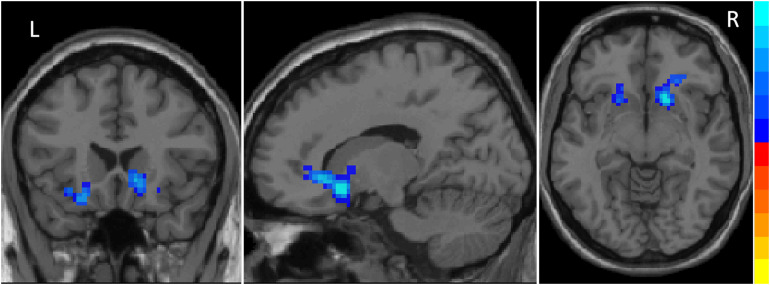
Decreased ReHo values in striatum including the right caudate, left pallidum and bilateral putamen in iRBD patients. The results were corrected by GRF (significant level is set at *P* < 0.05 at cluster and *P* < 0.01 at voxel level).

Compared to HC, iRBD group presented decreased ReHo in the right caudate, left pallidum, and bilateral putamen at the pre-established statistical threshold corrected by GRF (*P*_*voxel*_ < 0.01, *P*_*cluster*_ < 0.05; Figure 1). The peak MNI coordinates are listed in Table 2.

**TABLE 2:** Brain regions with altered ReHo in iRBD patients compared to control groups.

Brain region	MNI			Voxels	*t*-value
	*x*	*y*	*z*		
Caudate_R	14	23	−4	37	–4.6113
Putamen_L	−24	−6	6	80	–4.3597
Putamen_R	17	16	−8	19	–4.298
Pallidum_L	−18	3	−4	16	–3.422

*The significant threshold was defined as *P*_*cluster*_ < 0.05, *P*_*voxel*_ < 0.01, Gaussian random field (GRF) correction and cluster size >10 voxels. L, left; R, right; MNI, Montreal Neurological Institute; *x, y, z*, coordinates of primary peak locations in the MNI space.*

### Seed-Based Functional Connectivity Analysis

**FIGURE 2:**
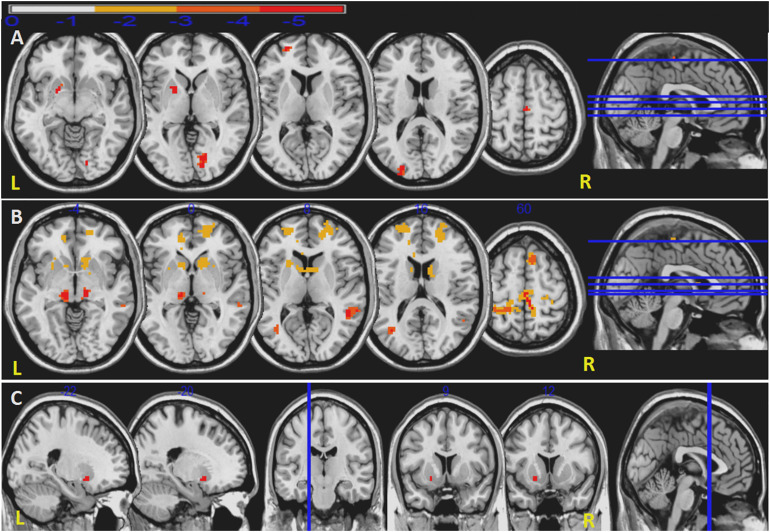
Decreased FCs in iRBD patients. Decreased right caudate-related FCs **(A)**. Decreased left pallidum-related FCs **(B)**. Decreased FCs between right putamen and left putamen **(C)**. Significant level is set at *P* < 0.05 corrected by TFCE.

Unlike priori choice of a brain area as seed for FC analysis, we selected seeds from ReHo results. This data-driven FC analysis has more theoretical basis and helps to explain the results. After the abnormal ReHo regions identified at the striatum, we set the right caudate, left pallidum and bilateral putamen as seeds to analyze striatum-related FCs. Compared to HC, in iRBD group, decreased right caudate-related FCs were seen in regions of the left superior frontal gyrus, left middle occipital gyrus, left pallidum, right precentral gyrus, right supplementary motor area (SMA), and right lingual gyrus. Decreased left pallidum-related FCs were seen in regions of left middle frontal gyrus, left Heschl’s gyrus; right SMA, right precentral gyrus, right paracentral lobule, right caudate; and bilateral superior frontal gyrus, middle temporal gyrus, anterior cingulate cortex, thalamus. Decreased intrinsic FC between bilateral putamen was also seen (Figure 2 and Table 3).

**TABLE 3:** Decreased functional connectivity in iRBD patients compared to controls.

Seeds	Brain region	MNI			Voxels	*t*-value
		*X*	*Y*	*Z*		
Caudate_R	Frontal_Sup_L	−24	54	9	35	−4.997^a^
	Supp_Motor_Area_R	3	−18	63	46	−5.0656^a^
	Occipital_Mid_L	−24	−96	15	108	−5.3639^a^
	Lingual_R	15	−75	0	196	−5.6011^a^
	Pallidum_L	−18	6	−3	163	−5.9774^a^
	Precentral_R	15	−21	78	11	−4.8479^a^
Pallidum_L	Frontal_Sup_L	−24	54	9	139	−4.9126^b^
	Frontal_Sup_R	21	45	18	34	−4.5608^b^
	Frontal_Mid_L	−30	42	24	25	−4.2613^b^
	Supp_Motor_Area_R	9	21	60	47	−4.6557^b^
	Precentral_R	24	−29	65	261	−4.6385^b^
	Paracentral_Lobule_R	6	−30	60	16	−4.2161^b^
	Temporal_Mid_L	−36	−66	12	109	−5.1764^b^
	Temporal_Mid_R	51	−45	6	128	−5.6246^b^
	Heschl_L	−60	−9	12	65	−4.5731^b^
	Cingulum_Ant_L	−12	45	0	21	−4.2566^b^
	Cingulum_Ant_R	12	39	9	12	−4.4244^b^
	Thalamus_L	−12	−21	−3	12	−5.3868^b^
	Thalamus_R	12	−21	−3	17	−4.2929^b^
	Caudate_R	12	15	0	83	−4.5599^b^
Putamen_R	Putamen_L	−21	9	−9	7	−5.8556^b^

*The significant threshold was defined as *P* < 0.05, Threshold-free cluster enhancement (TFCE) correction and cluster size >10 voxels. ^a^*P* < 0.03; ^b^*P* < 0.05. L, left; R, right; MNI, Montreal Neurological Institute; x, y, z, coordinates of primary peak locations in the MNI space.*

### Correlation Between Functional Connectivity and Cognitive Performance

**FIGURE 3:**
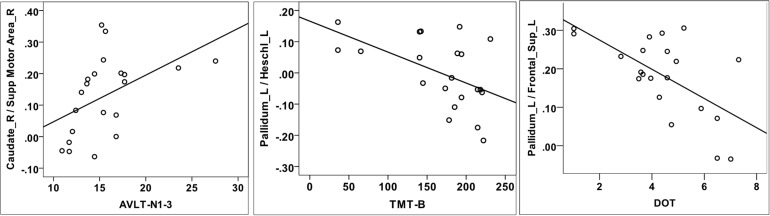
Correlation analysis between decreased FCs and cognitive tests. AVLT-N1-3 scores were positively correlated with FCs between right caudate and right supplementary motor area **(A)**. TMT-B scores were negatively correlated with FCs between left pallidum and left Heschl’s gyrus **(B)**. DOT scores were negatively correlated with FCs between left pallidum and left superior frontal gyrus **(C)**.

No significant associations were found between abnormal ReHo regions and cognitive subtest scores (ROCFT_copy, AVLT-N1-3, TMT-B, DOT, and SDMT). Then, we aimed to verify whether the attention/working memory and executive deficits found in the neuropsychological test were correlated with the striatal-cortical network changes in RBD patients. Correlation analyses were carried out between decreased FCs and cognitive subtests scores with age, education, and sex adjusted. AVLT-N1-3, TMT-B, and DOT scores were associated with the decreased FC. TMT-B scores were negatively correlated with FCs between left pallidum and left Heschl’s gyrus (*r* = −0.500, *P* = 0.035), left pallidum, and left middle temporal gyrus (*r* = −0.484, *P* = 0.042). DOT scores were negatively correlated with left pallidum-related FCs to the left Heschl’s gyrus (*r* = −0.444, *P* = 0.042), left middle frontal gyrus (*r* = −0.520, *P* = 0.027), right caudate (*r* = −0.490, *P* = 0.039), right anterior cingulate cortex (*r* = −0.482, *P* = 0.043), and the bilateral superior frontal gyrus (*r* = −0.444, *P* = 0.042). AVLT-N1-3 were positively correlated with FCs between right caudate and right SMA (*r* = 0.474, *P* = 0.047), left pallidum, and right middle temporal gyrus (*r* = 0.530, *P* = 0.024). Figure 3 shows three representative results of the correlation analysis between FCs and cognitive subtests.

## Discussion

In the present study, we used both ReHo and seed-based FC approaches to explore the FC abnormalities and its association with cognitive function iRBD patients. First, iRBD patients presented cognitive deficits in attention/working memory, executive function, immediate memory, and visuo-spatial ability domains. Second, ReHo analysis revealed abnormal spontaneous brain activities in the striatum (right caudate, left pallidum, and bilateral putamen) in iRBD patients. Third, iRBD patients showed decreased striatum-related FCs to the frontal, temporal, occipital lobes, thalamus, and anterior cingulate gyrus, as well as decreased intrinsic FCs between bilateral striatums. Finally, attention, executive function, and immediate memory were associated with abnormal striatal-cortical FCs including frontal, temporal, and anterior cingulate cortices.

The present study detected decreased ReHo in the striatum including caudate, pallidum and putamen in iRBD patients, which added further evidence that the striatum was damaged in iRBD. Previous study reported similar results that ReHo was decreased in putamen in iRBD. Besides, they found a positive correlation between the decreased ReHo and decreased dopamine transporter in the same region (Li et al., 2020). Several studies have shown that iRBD patients exhibited striatum volume changes, abnormal glucose metabolism and nigrostriatal dopaminergic dysfunction (Ellmore et al., 2010; Vendette et al., 2011; Dang-Vu et al., 2012; Bauckneht et al., 2018; Rahayel et al., 2018a). Early alterations of glucose metabolism, dopamine transporter, and functional activities by ReHo analysis preceded the subsequent volume changes. However, both glucose metabolism and dopamine transporter are detected by PET-CT or SPECT that are expensive and radioactive. Thus, abnormal ReHo may serve as an alternative neuroimaging biomarker for iRBD monitoring.

On FC analysis, we found decreased striatum-related FCs in motor-related frontal cortices such as precentral gyrus and SMA. Meanwhile, intrinsic FCs were also decreased between the striatum, which were involved in production and regulation of motor network (Burciu and Vaillancourt, 2018). One previous study reported similar decreased intrinsic FCs between striatum in both iRBD and PD patients (Li et al., 2020). In *de novo* PD patients, there were similar decreased FCs between the striatum and motor-related frontal lobe (Esposito et al., 2013). Based on the above findings, decreased FCs between the striatum and motor-related frontal lobe may be the neural basis for future motor impairment in iRBD.

On neuropsychological tests, iRBD patients usually exhibit multiple cognitive domain impairments, but language is relatively spared. One study found decreased visual discrimination iRBD was associated with hippocampal hyperperfusion (Dang-Vu et al., 2012). In another study, the volumes of frontal, temporal, parietal, occipital, striatum, and thalamus were decreased in iRBD patients with cognitive impairment (Rahayel et al., 2018b). The neocortex, limbic lobe, striatum, and thalamus seemed all involved in the cognitive impairment of iRBD (Ferini-Strambi et al., 2019). Nevertheless, the cognitive impairment in iRBD were seldomly investigated by the method of FC, which had an advantage in revealing brain cognitive function networks. Previously only one study found thalamo-occipital FC was associated with the cognitive function in iRBD by defining thalamus as the seed (Byun et al., 2020).

Under physiological conditions, the basal ganglia form three dopaminergic circuits *via* thalamus to the cortex, that are, motor circuit, associative circuit, and limbic circuit (Krack et al., 2010). In PD, in addition to the motor circuit, the associative and limbic circuits are also affected. The associative circuit connects the basal ganglia to the dorsolateral prefrontal and lateral orbitofrontal cortices, which are involved in cognitive processes such as working memory and executive function. In the current study, we found that abnormal striatal-frontal FC in RBD was correlated with attention/working memory. In detail, AVLT-N1-3 scores were positively associated with some striatal-cortical functional connections, while TMT-B and DOT scores were negatively associated with some other striatal-cortical functional connections. In other words, reduced striatal-frontal FC corresponds to worse attention and executive function. However, reduced striatal-frontal FC corresponds to relatively better working memory, which seems difficult to explain. As working memory declined in the RBD group, one possible explanation is that the striatal-frontal FC is enhanced to compensate for the declined working memory. We also found AVLT-N1-3 was correlated with FC between right caudate and right SMA. AVLT-N1-3 documents short-term memory, also indicating attention and working memory. Through the investigation of patients with SMA lesions, there have been some studies revealing SMA lesions could impair the working memory (Nakajima et al., 2014; Canas et al., 2018).

Although the pathology of iRBD started in the lower brainstem (usually Lewy body deposition), functional or metabolic abnormalities could already occur in the basal ganglia, which partially explained the early cognitive impairment in RBD (Chung et al., 2018). Over time, the involvement of the basal ganglia became more pronounced, finally leading to motor symptoms. Due to the individual differences in Lewy body deposition and spreading, cognitive impairment in iRBD may be heterogeneous and multiple neural networks involved (Ferini-Strambi et al., 2019).

There were some limitations in our study. First, the language domain subtest was not included in our study. Some studies suggested that language function was least affected in iRBD. Second, the present study was a cross-sectional design. Future longitudinal investigations may provide insight into cognitive changes over time and causality between cognition decline and neural network abnormalities. Besides, sample size was limited.

In conclusion, functional changes of striatum and cognitive impairment in iRBD were reconfirmed in the present study. The impairment of working memory/executive function in iRBD was related to the striatal-cortical network, especially the striatal-frontal network. These findings supported the role of striatum not only in motor but also in cognition impairment in iRBD.

## Data Availability Statement

The raw data supporting the conclusions of this article will be made available by the authors, without undue reservation.

## Ethics Statement

The studies involving human participants were reviewed and approved by Medical Ethics Committee of People’s Hospital of Zhengzhou University. The patients/participants provided their written informed consent to participate in this study.

## Author Contributions

SC and HZ: conceptualization, writing, and resources and supervision. SW and YB: methodology. JZ: data analysis. All authors contributed to the article and approved the submitted version.

## Conflict of Interest

The authors declare that the research was conducted in the absence of any commercial or financial relationships that could be construed as a potential conflict of interest.

## Footnotes

1 http://rfmri.org/dparsf

2 http://www.fil.ion.ucl.ac.uk/spm

3 http://www.restfmri.net/
